# Are more physical education classes related to less time in leisure-time sedentary behavior? An analysis including adolescents from 73 countries

**DOI:** 10.1186/s12889-023-16703-7

**Published:** 2023-10-07

**Authors:** Danilo R. Silva, Raphael H. O. Araujo, André O. Werneck, Giada Ballarin, Federica Andricciola, Leandro dos Santos, Javier Brazo-Sayavera

**Affiliations:** 1 Faculty of Health Sciences, Universidad Autónoma de Chile, Providencia, Chile; 2 Department of Sports and Computer Science, Universidad Pablo de Olavide (UPO), 41013 Seville, Spain; 3 Graduate Program in Health Sciences, Londrina State University, Londrina, Brazil; 4 Center for Epidemiological Research in Nutrition and Health, Department of Nutrition, School of Public Health, Universidade de São Paulo (USP), São Paulo, Brazil; 5 Physical Activity, Health and Sport Research Group, Universidad Pablo de Olavide (UPO), 41013 Seville, Spain; 6 Department of Movement Sciences and Wellbeing, University of Naples “Parthenope”, Naples, Italy; 7 Department of Health Sciences, University of “Magna Græcia” of Catanzaro, Catanzaro, Italy

**Keywords:** Health Behavior, Health Education, School Health Services

## Abstract

This study aimed to verify the association between the weekly frequency of physical education (PE) classes and leisure sitting time among adolescents. We analyzed data from 73 countries using the Global School-based Student Health Survey (283,233 adolescents between 11 and 18 years of age). Leisure sitting time and weekly frequency of PE classes were self-reported. Sex, age, and food insecurity were used as covariates and the analyses were stratified by world regions and country income level. Poisson regression models (random-effects meta-analysis) were used for the main analyses. In comparison with 1 PE class per week (reference group), those with no PE classes presented a lower prevalence of ≥ 3 h/d of leisure sitting time (PR [95%CI] = 0.94 [0.91; 0.98]). On the other hand, adolescents with 2 days (PR [95%CI] = 1.06 [1.02; 1.26]), 3–4 days (PR [95%CI] = 1.17 [1.12; 1.22]), and 5 days (PR [95%CI] = 1.08 [1.04; 1.11]) of PE classes presented a higher likelihood of ≥ 3 h/d of leisure sitting time. No clear differences were observed for the different world regions and country income levels. We conclude that a higher weekly frequency of PE classes is associated with increased leisure sitting time among adolescents worldwide.

**Supplementary Information:**

The online version contains supplementary material available at 10.1186/s12889-023-16703-7.

## Introduction

Sedentary behavior represents a public health concern [1]. Among school-aged children and adolescents, sedentary behavior is associated with cardiometabolic, social, and mental health-related outcomes [2]. Similarly to other health-related behaviors, preventive actions should be developed since early ages. In addition, there is evidence that sedentary behaviors increase substantially from school age [3]. Thus, increased attention is being paid to sedentary behavior at school [4], as well as to how school-based approaches may help in the promotion of an active lifestyle, including sustainable behaviors outside school [5].

Curricular physical education (PE) is a school-based opportunity to influence movement behaviors as it has the role of making physical activity a meaningful component of a student’s life, with the objective of both improving physical activity and understanding other aspects of the students’ lives, including other health-related behaviors [6]. Previous evidence suggests that a higher weekly frequency of PE classes is related to a higher physical activity level in children and adolescents [7, 8], among other benefits, such as academic performance and cognition [9]. However, the relationship between the frequency of PE classes and sedentary time is still unclear.

Systematic reviews identified inconsistent associations between the frequency of PE classes and sedentary behaviors [7, 8]. Studies comparing days with and without PE classes showed that less sitting time was observed on PE class days [10, 11]; however, this relationship was not clear when analyzing the usual sedentary behavior, including weekend days [12, 13]. In addition, previous investigations were mostly based on specific countries, and only a few were based on representative samples and with different proxies of sedentary behavior, such as screen time or school time. More recently, Martins et al. [14] explored the association between the frequency of PE classes and general health-related behaviors in a worldwide database and observed intriguing findings on a positive association between the frequency of PE classes and leisure sitting time. However, the authors used the category of no PE class as the reference group, which could include a specific group of the population, as PE classes are mandatory in most countries. In addition, the differences between countries were not explored. It could be hypothesized that given the differences in the access and opportunities for an active lifestyle [15], its role and association with the frequency of PE classes would differ between the world regions and according to the level of income in the country.

Therefore, a comprehensive and deeper worldwide view of the association between the frequency of PE classes and leisure sitting time is lacking. Clarifying this relationship could help policymakers to reinforce the role and frequency of curricular PE on health-behaviors outside school hours, as well as providing elements that enable basis of the PE curriculum to be rethought, focusing on optimizing its impact on the life of young people. Thus, the current study aims to verify the association between the number of PE classes and leisure sitting time among adolescents worldwide.

## Methods

### Design and sample

This is a multi-country cross-sectional study based on the Global School-based Student Health Survey (GSHS) database. The GSHS was conducted in 104 countries with a standard school-based sampling process and a core self-administered questionnaire [16]. The specific procedures of data collection were approved by local ethics committees and the consent form was signed by school staff, students, and their legal guardians.

For the current study, we included the most recent survey from 73 countries (only those with national coverage) with available data on PE classes, leisure sitting time, and covariates. The included surveys were conducted between 2009 and 2018, with participants’ ages ranging between 11 and 18 years, totaling 283,233 participants. Further details about each survey are presented in Supplementary table 1.

### Sedentary behavior

Leisure sitting time, as a proxy of sedentary behavior, was assessed through the question: “*When you are not at school or doing homework, how much time do you spend during a typical or usual day sitting and watching television, playing computer games, talking with friends, or doing other sitting activities?*”. Possible answers were: < 1 h/d, 1 to 2 h/d, 3 to 4 h/d, 5 to 6 h/d, 7 to 8 h/d, and > 8 h/d. We used the cutoff of ≥ 3 h/d to classify leisure sitting time based on previous GSHS studies [14, 17]. However, for sensitivity analysis purposes, we present the main results using ≥ 5 h/d as the cutoff in Supplementary table 4.

### Physical education classes

The weekly frequency of physical education class attendance was assessed through the question: “*During this school year, on how many days did you go to physical education classes each week?*”. Possible answers were 0, 1, 2, 3, 4, 5, or more days per week. Considering the low frequency of 3 (4.5%) and 4 (2.9%) days, we joined these categories for the main analyses.

### Covariates and contextual variables

Sex (male and female), age group (11–14 and 15–18), and food insecurity (i.e., a proxy for socioeconomic status) were used as covariates. We considered as food insecurity the frequency (sometimes/most of the time/always) of hunger due to lack of food, based on the question: “*During the past 30 days, how often did you go hungry because there was not enough food in your home?*”.

For analyses purpose, countries were divided into five regions (East Asia & Pacific, Latin America & Caribbean, Middle East & North Africa, South Asia, Sub-Saharan Africa) and income levels (low, lower-middle, upper-middle, high) [18].

### Statistical analyses

Data are described as absolute and relative frequencies as well as confidence intervals of 95% (95%CI). Prevalence was pooled using proportional metanalyses models. Poisson regression models were used to estimate the association between PE classes and leisure sitting time, and the prevalence ratios (RP) were pooled through random-effects meta-analysis models, according to region and income level. Regression analyses were adjusted for sex, age group, and food insecurity. All analyses were conducted in Stata 15.0 software.

## Results

The characteristics of the sample are presented in Table 1. A total of 283,233 participants from 73 countries were included. Half of the sample were girls. Although ages ranged between 11 and 18 among the surveys, the proportion of younger adolescents (11–14 year-old) varied between 36% (Sub-Saharan Africa) and 53% (Middle East & North Africa and South Asia). In general, 75% of the sample had at least one PE class per week, with no clear differences between countries. The prevalence of ≥ 3 h/d of leisure sitting time varied between 19% (South Asia) and 48% (Latin America & Caribbean) among regions and increased according to the income level of the country.

**Table 1:** Characteristics of the sample

	Number of countries	Number of participants	% Girls	% 11–14 y	% Food insecurity	% ≥ 1 PE d/wk	% ≥ 3 h/d of LST
**Total**	73	283,233	50 (49; 51)	47 (44; 51)	28 (25; 31)	75 (72; 77)	38 (34; 42)
**Region**							
East Asia & Pacific	21	85,157	51 (50; 52)	44 (37; 51)	37 (32; 42)	76 (72; 81)	32 (25; 38)
Latin America & Caribbean	26	110,005	51 (51; 52)	50 (45; 54)	21 (18; 24)	77 (73; 80)	48 (43; 53)
Middle East & North Africa	12	45,775	50 (48; 51)	53 (44; 63)	24 (20; 29)	72 (66; 78)	40 (31; 48)
South Asia	5	19,151	44 (38; 51)	53 (44; 62)	31 (19; 42)	67 (50; 84)	19 (11; 28)
Sub-Saharan Africa	9	23,145	47 (41; 53)	36 (24; 48)	34 (24; 44)	73 (64; 81)	31 (24; 39)
**Income**							
Low	6	13,406	47 (45; 48)	39 (20; 59)	38 (27; 49)	67 (52; 81)	25 (20; 29)
Lower-middle	28	100,676	49 (47; 51)	48 (41; 55)	30 (25; 34)	76 (71; 81)	28 (24; 33)
Upper-middle	22	123,357	51 (50; 52)	45 (41; 49)	28 (22; 33)	73 (69; 77)	39 (34; 45)
High	17	45,794	52 (51; 52)	52 (44; 60)	23 (18; 27)	78 (73; 82)	56 (52; 59)

Note. *PE* Physical Education classes. *LST* Leisure sitting time. Values expressed in prevalence (95% confidence interval)

**Fig. 1:**
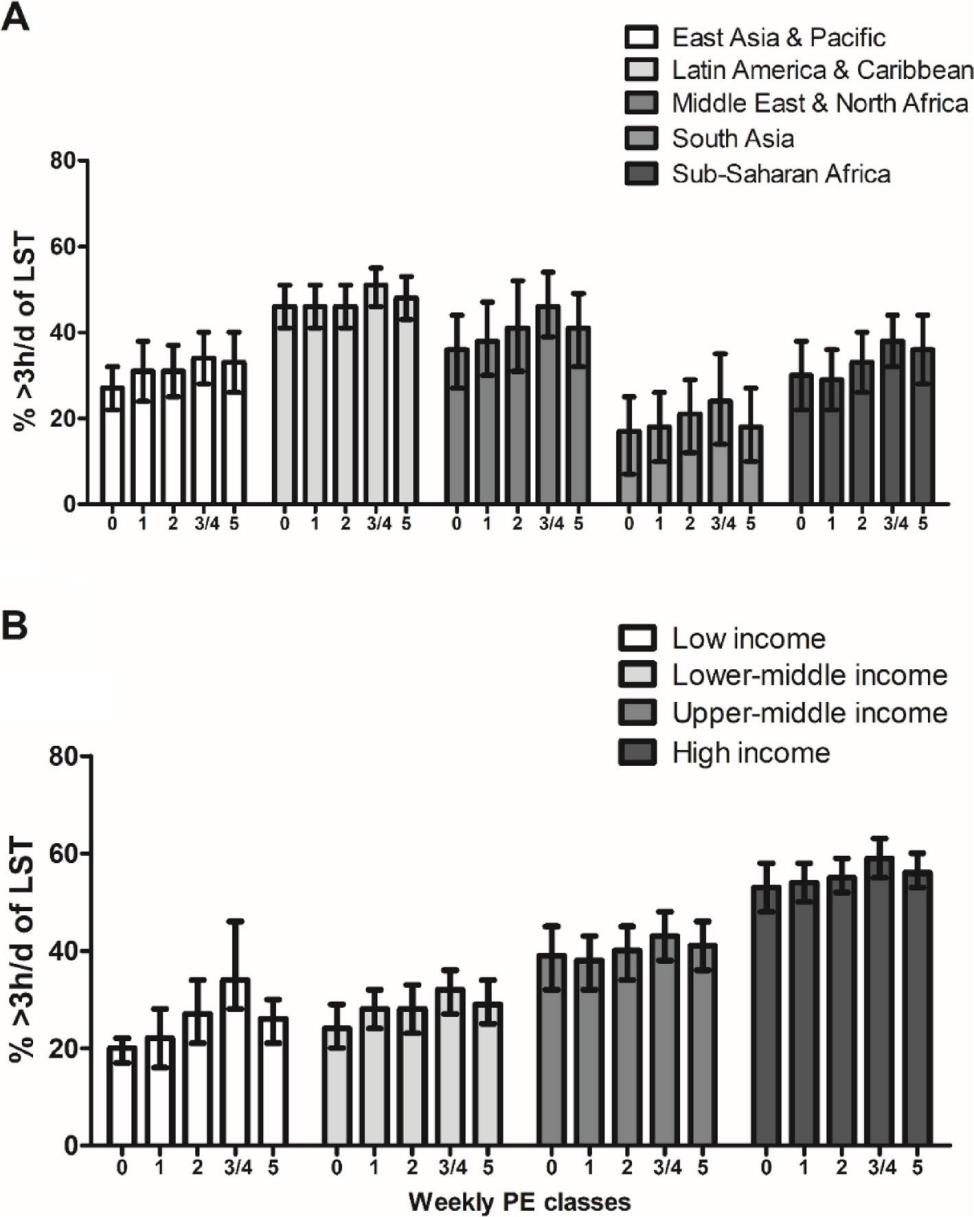
Prevalence (%) of ≥ 3 h/d of leisure sitting time (LST) according to the number of weekly PE classes by region and income. Note. *PE* Physical Education. Values expressed in prevalence (95% confidence interval)

A non-linear association was observed between weekly PE classes and leisure sitting time, where both extreme groups (0 and ≥ 5 weekly classes) tended to show lower leisure sitting time (Fig. 1). Supplementary Table 2 presents the prevalence of ≥ 3 h/d of leisure sitting time according to the weekly frequency of PE classes by country.

The pooled adjusted regression models considering 1 day of PE classes per week as a reference are presented in Table 2. For the total sample, we observed that no PE classes was associated with lower leisure sitting time and more than one day per week was associated with higher leisure sitting time. Similar patterns were observed within regions and income levels. A higher likelihood of ≥ 3 h/d leisure sitting time was observed when PE classes took place 3–4 days per week. South Asia was the only region that did not show significant associations. Supplementary Table 3 presents the specific associations by country. A sensitivity analysis adopting ≥ 5 h/d of leisure sitting time as the cutoff point is presented in Supplementary Table 4, where we also observed increased leisure sitting time with a higher frequency of PE classes, especially from 3–4 PE classes per week.

**Table 2:** Association between the number of PE classes and leisure sitting time (≥ 3 h/d) according by region and income

	**Number of weekly PE classes**				
	0 day	1 day	2 days	3–4 days	≥ 5 days
**Total**	0.94 (0.91; 0.98)	REF	1.06 (1.02; 1.26)	1.17 (1.12; 1.22)	1.08 (1.04; 1.11)
**Region**					
East Asia & Pacific	0.87 (0.79; 0.96)	REF	1.01 (0.95; 1.08)	1.15 (1.03; 1.27)	1.08 (0.98; 1.18)
Latin America & Caribbean	0.97 (0.92; 1.02)	REF	1.05 (1.01; 1.10)	1.11 (1.06; 1.16)	1.04 (1.00; 1.07)
Middle East & North Africa	0.94 (0.87; 1.02)	REF	1.06 (0.99; 1.13)	1.18 (1.10; 1.26)	1.07 (1.01; 1.12)
South Asia	0.92 (0.75; 1.16)	REF	1.16 (0.86; 1.57)	1.35 (0.95; 1.93)	1.01 (0.79; 1.30)
Sub-Saharan Africa	1.05 (0.94; 1.17)	REF	1.16 (1.08; 1.26)	1.33 (1.19; 1.47)	1.26 (1.12; 1.43)
**Income**					
Low	0.94 (0.84; 1.05)	REF	1.26 (0.99; 1.60)	1.56 (1.26; 1.94)	1.18 (1.00; 1.38)
Lower-middle	0.89 (0.81; 0.98)	REF	1.03 (0.96; 1.09)	1.19 (1.09; 1.30)	1.05 (0.98; 1.14)
Upper-middle	0.99 (0.93; 1.07)	REF	1.07 (1.02; 1.13)	1.15 (1.07; 1.24)	1.11 (1.04; 1.18)
High	0.96 (0.92; 1.01)	REF	1.05 (1.01; 1.10)	1.11 (1.06; 1.16)	1.05 (1.02; 1.09)

Note. *REF* Reference group. Values expressed in prevalence ratio and 95% confidence interval. Adjusted for age, sex and food insecurity

## Discussion

Our main finding was that the increased weekly frequency of PE classes was associated with higher leisure sitting time. While these results were not expected, they should be interpreted based on their context and methodological issues. Although many confounders could explain these cross-sectional associations, the findings show that a higher number of PE classes per se, despite being positive for many other outcomes, was not associated with leisure sitting time among adolescents around the world.

Based on the role of the curricular PE, it could be expected that a higher weekly number of PE classes would be related to a more active and less sedentary lifestyle. In fact, evidence shows that children and adolescents exposed to a higher frequency of PE classes are more active [7, 8]; however, achieving physical activity guidelines or performing more moderate and vigorous physical activity does not mean being less sedentary [1]. In this sense, although accelerometer-based studies showed that adolescents spent less overall sedentary time on days with a PE class [10, 11], this is not clear when considering the whole week or specifically leisure sitting time [12]. Thus, the current findings indicate a positive relationship between the frequency of PE classes and a general indicator of leisure sedentary behaviors with a basis in a worldwide sample.

Some reasons could explain these results. First, the “ActivityStat” hypothesis suggests that people tend to compensate for higher energy expenditure in a specific domain of the day with less physical activity in other domains of the day [19]. Given that adolescents who have more frequent PE classes tend to be more active (both at school and outside school hours), they could compensate for this time with highly sedentary behavior for the rest of the day or week. Second, both the frequency of PE classes and the access/opportunities for sedentary behaviors (e.g., technology-based devices) may be higher in the wealthiest groups. This could also explain the lower leisure sitting time among the groups without PE classes, which could occur in schools with low resources [20]. Thus, although we did not observe significant differences between regions or income levels, these differences can exist within countries. Lastly, the content of classes, PE curricular guidelines as well as the education, knowledge and competences of PE teachers were not considered, which can affect how PE classes could be related to health behaviors outside school (e.g. approaching “sedentary behavior” or “screen time” as specific behaviors). This is a possible and understandable hypothesis, especially considering that the evidence on sedentary behavior as an independent public health issue is more recent in comparison with physical inactivity. Further studies are needed to clarify these hypotheses.

In practical terms, our results indicate that further efforts should be made to reduce leisure-time sedentary behaviors among children and adolescents. Education for health must receive attention in schools to expand the school’s borders in favor of youth health and development. PE can increase physical activity enjoyment and motor competence, which could predict less sedentary lifestyle [21]. Potential avenues to reduce should acting on the determinants of leisure-time sedentary behaviors, involving families, and using technology (e.g., apps with gamification tools and screen time goals) [5, 22].

Our study has limitations that should be mentioned. First, due to its cross-sectional design and the lack of control for potential confounders (e. g., specific legislation, contents and the teaching methodology of PE classes between and within countries, country culture issues, household environment, type of school, socioeconomic status, maternal education, etc.), causal inference between PE classes and leisure sitting time is not possible. Second, self-reported PE classes and leisure sitting time can contain understanding and recall bias. Future studies are needed to clarify how PE classes are associated with different domains of leisure sitting time (e.g., reading, TV or smartphone time) and the use of questionnaires in association with device-based measures is recommended. Third, we adopted 3 h/d as a cutoff point for leisure sitting time based on previous GSHS studies [14, 17] as well as the sample distribution. Although this seems a relatively low cutoff considering the whole leisure-time sedentary behavior (for example, 2 h/d has been recommended for recreational screen time only [23]), adolescents tend to underestimate their sitting time in self-report methods [24, 25]. Therefore, we can speculate that the cutoff point used could represent more than 3 h/d. In any case, the sensitivity analysis using ≥ 5 h/d as a cutoff presents similar results, and even stronger associations, compared to the use of ≥ 3 h/d, which reinforce the direction of the associations found. Lastly, although we tested gradual relationships using the association with the different numbers of PE class days, it was not possible to consider 3 and 4 days separately due to the reduced sample size. However, we provide evidence from a large worldwide sample based on a representative sample of more than 70 countries, which strengthens the external validity and comprehensiveness of the current findings.

In conclusion, a higher weekly frequency of PE classes is associated with increased leisure sitting time. Further prospective studies should explore details about the PE classes (e.g. curricular guidelines, teaching methodology), using device-based methods in association with self-reported questionnaires to measure different aspects of the sedentary time during the week and weekend. This approach can clarify the potential causal pathways between these variables.

### Supplementary Information

**Additional file 1: Supplementary table 1.** Characteristics of the sample by country (n = 283,233). Note. PE, Physical Education classes. LST, Leisure sitting time. Values expressed in prevalence (95% confidence interval). **Supplementary table 2. **Prevalence (%) of ≥3h/d of leisure sitting time according the number of weekly PE by country. Note. PE, Physical Education. Values expressed in prevalence (95% confidence interval).**Supplementary table 3. **Association between the number weekly PE classes and leisure sitting time (≥3 h/d) by country.Note. REF, Reference group. PE, Physical Education. Values expressed in prevalence ratio and 95% confidence interval. Adjusted for age, sex and food insecurity. **Supplementary table 4.** Association between the number of PE classes and leisure sitting time (≥5 h/d) according by region and **income. Note. RE**F, Reference group. Values expressed in prevalence ratio and 95% confidence interval. Adjusted for age, sex and food insecurity.

## Data Availability

The Global School-based Student Health Survey database is open and can be accessed in: https://www.cdc.gov/gshs/index.htm
