# Supplementary material for: Are more physical education classes related to less time in leisure-time sedentary behavior? An analysis including adolescents from 73 countries
Source: BMC Public Health. 2023 Oct 7;23:1943. doi: 10.1186/s12889-023-16703-7 (PMC10559448; doi:10.1186/s12889-023-16703-7)
Supplement: Supplementary file 1 — Additional file 1: Supplementary table 1. Characteristics of the sample by country (n = 283,233). Note. PE, Physical Education classes. LST, Leisure sitting time. Values expressed in prevalence (95% confidence interval). Supplementary table 2. Prevalence (%) of ≥3h/d of leisure sitting time according the number of weekly PE by country. Note. PE, Physical Education. Values expressed in prevalence (95% confidence interval).Supplementary table 3. Association between the number weekly PE classes and leisure sitting time (≥3 h/d) by country.Note. REF, Reference group. PE, Physical Education. Values expressed in prevalence ratio and 95% confidence interval. Adjusted for age, sex and food insecurity. Supplementary table 4. Association between the number of PE classes and leisure sitting time (≥5 h/d) according by region and income. Note. REF, Reference group. Values expressed in prevalence ratio and 95% confidence interval. Adjusted for age, sex and food insecurity. [file 12889_2023_16703_MOESM1_ESM.docx]

**Supplementary table 1.** Characteristics of the sample by country (n = 283,233).

| Region/Country | Year | Response rate (%) | n final | % Girls | % 11-14 y | % Food insecurity | % ≥1 PE d/wk | % ≥ 3h/d of LST |
| --- | --- | --- | --- | --- | --- | --- | --- | --- |
| **East Asia & Pacific** |  |  |  |  |  |  |  |  |
| Brunei Darussalam | 2014 | 65 | 2501 | 50.3 | 48.8 | 32.0 | 79.4 | 56.2 |
| Cambodia | 2013 | 85 | 3710 | 48.0 | 32.9 | 35.3 | 63.8 | 11.9 |
| Cook Islands | 2015 | 65 | 684 | 51.1 | 32.1 | 43.6 | 71.4 | 44.4 |
| Fiji | 2016 | 79 | 3409 | 51.2 | 22.4 | 50.3 | 80.1 | 29.1 |
| Indonesia | 2015 | 94 | 10778 | 51.1 | 67.6 | 43.3 | 87.7 | 27.2 |
| Kiribati | 2011 | 85 | 1506 | 53.9 | 56.0 | 42.7 | 78.5 | 14.9 |
| Laos | 2015 | 70 | 3610 | 47.0 | 15.9 | 33.1 | 66.7 | 21.1 |
| Malaysia | 2012 | 89 | 25220 | 49.9 | 41.7 | 31.5 | 88.9 | 47.3 |
| Mongolia | 2013 | 88 | 5255 | 51.6 | 49.3 | 14.2 | 95.3 | 44.4 |
| Myanmar | 2016 | 86 | 2711 | 53.8 | 67.6 | 24.6 | 70.1 | 15.8 |
| Philippines | 2015 | 79 | 136 | 42.4 | 47.4 | 38.5 | 67.3 | 33.8 |
| Samoa | 2017 | 79 | 8600 | 50.7 | 49.8 | 38.1 | 86.0 | 31.3 |
| Solomon Islands | 2011 | 85 | 1724 | 54.2 | 40.2 | 37.9 | 45.3 | 27.1 |
| Thailand | 2015 | 89 | 1257 | 46.5 | 43.1 | 75.3 | 72.6 | 25.0 |
| Timor-Leste | 2015 | 79 | 2418 | 54.8 | 51.0 | 29.8 | 86.4 | 56.8 |
| Tonga | 2017 | 90 | 3292 | 49.8 | 24.4 | 37.5 | 83.7 | 14.3 |
| Tuvalu | 2013 | 90 | 125 | 45.9 | 60.7 | 45.7 | 80.6 | 48.5 |
| Vietnam | 2013 | 96 | 3189 | 49.5 | 56.1 | 40.6 | 49.1 | 20.8 |
| Wallis and Futuna | 2015 | 82 | 831 | 52.2 | 58.9 | 23.7 | 56.2 | 17.1 |
| **Latin America & Caribbean** |  |  |  |  |  |  |  |  |
| Anguilla | 2016 | 88 | 760 | 51.8 | 46.0 | 23.5 | 84.1 | 59.5 |
| Antigua and Barbuda | 2009 | 67 | 1048 | 48.4 | 68.2 | 29.9 | 72.9 | 55.1 |
| Argentina | 2018 | 71 | 53483 | 52.3 | 45.6 | 11.4 | 88.0 | 55.5 |
| Bahamas | 2013 | 78 | 1253 | 52.5 | 83.1 | 29.0 | 92.1 | 54.6 |
| Barbados | 2011 | 73 | 1541 | 51.0 | 58.4 | 23.0 | 82.9 | 65.4 |
| Belize | 2011 | 88 | 1995 | 52.4 | 63.4 | 25.3 | 67.7 | 37.5 |
| Bolivia | 2018 | 88 | 7089 | 49.1 | 30.1 | 20.4 | 88.6 | 31.2 |
| British Virgin Islands | 2009 | 90 | 1569 | 53.3 | 57.0 | 25.0 | 62.9 | 61.5 |
| Chile | 2013 | 60 | 1900 | 51.5 | 40.1 | 8.1 | 91.3 | 54.0 |
| Costa Rica | 2009 | 72 | 2597 | 49.5 | 56.4 | 7.7 | 79.9 | 43.6 |
| Curaçao | 2015 | 83 | 2551 | 52.1 | 31.3 | 13.0 | 79.1 | 62.1 |
| Dominican Republic | 2016 | 63 | 1344 | 50.9 | 25.7 | 14.9 | 74.8 | 46.9 |
| El Salvador | 2013 | 88 | 1789 | 48.5 | 58.6 | 14.3 | 87.1 | 34.5 |
| Guatemala | 2015 | 82 | 3814 | 47.5 | 56.7 | 13.9 | 89.3 | 22.4 |
| Guyana | 2010 | 76 | 2268 | 51.6 | 55.6 | 33.1 | 50.2 | 35.7 |
| Honduras | 2012 | 79 | 1666 | 53.4 | 68.1 | 13.8 | 90.7 | 30.2 |
| Jamaica | 2017 | 72 | 1558 | 51.7 | 31.0 | 28.2 | 58.1 | 56.9 |
| Panama | 2018 | 71 | 2770 | 52.8 | 33.1 | 16.8 | 73.1 | 48.3 |
| Paraguay | 2017 | 87 | 2933 | 51.6 | 42.4 | 11.6 | 84.7 | 34.4 |
| Peru | 2010 | 85 | 2850 | 49.5 | 51.9 | 19.2 | 92.2 | 28.7 |
| St. Kitts and Nevis | 2011 | 70 | 1632 | 49.6 | 50.1 | 26.4 | 62.1 | 59.2 |
| St. Lucia | 2018 | 77 | 1849 | 53.2 | 53.2 | 29.5 | 58.6 | 56.2 |
| St. Vincent and the Grenadines | 2018 | 78 | 1750 | 51.3 | 30.2 | 34.4 | 54.7 | 55.1 |
| Suriname | 2016 | 83 | 2038 | 51.1 | 49.0 | 32.8 | 79.4 | 42.0 |
| Trinidad and Tobago | 2017 | 89 | 2662 | 53.0 | 55.0 | 28.7 | 62.7 | 48.4 |
| Uruguay | 2012 | 77 | 3296 | 54.2 | 53.1 | 7.3 | 87.4 | 58.8 |
| **Middle East & North Africa** |  |  |  |  |  |  |  |  |
| Algeria | 2011 | 98 | 4364 | 52.2 | 57.5 | 30.4 | 83.5 | 27.1 |
| Bahrain | 2016 | 89 | 6997 | 49.6 | 54.5 | 33.5 | 87.4 | 58.4 |
| Egypt | 2011 | 85 | 2369 | 50.5 | 80.5 | 21.2 | 64.7 | 27.8 |
| Iraq | 2012 | 88 | 1871 | 42.9 | 50.9 | 16.7 | 64.5 | 26.3 |
| Kuwait | 2015 | 78 | 3189 | 49.5 | 35.9 | 26.5 | 81.4 | 64.6 |
| Lebanon | 2017 | 87 | 5175 | 54.7 | 49.2 | 11.8 | 58.6 | 44.9 |
| Morocco | 2016 | 92 | 5890 | 46.2 | 43.0 | 21.5 | 80.3 | 32.1 |
| Oman | 2015 | 92 | 3295 | 50.8 | 29.3 | 19.8 | 77.8 | 39.8 |
| Qatar | 2011 | 87 | 1657 | 53.0 | 83.7 | 15.8 | 70.5 | 47.4 |
| Syrian Arab Republic | 2010 | 97 | 3015 | 49.1 | 75.9 | 32.2 | 79.1 | 25.5 |
| United Arab Emirates | 2016 | 80 | 5591 | 50.9 | 38.7 | 27.1 | 73.6 | 59.2 |
| Yemen. Rep. | 2014 | 75 | 2362 | 45.2 | 41.3 | 31.9 | 46.4 | 21.9 |
| **South Asia** |  |  |  |  |  |  |  |  |
| Afghanistan | 2014 | 79 | 2148 | 44.1 | 35.7 | 38.5 | 68.4 | 24.5 |
| Bangladesh | 2014 | 91 | 2729 | 35.0 | 65.7 | 53.5 | 89.5 | 15.4 |
| Nepal | 2015 | 69 | 6079 | 51.2 | 55.8 | 27.4 | 66.8 | 10.6 |
| Pakistan | 2009 | 76 | 5025 | 39.1 | 61.7 | 19.2 | 40.7 | 8.1 |
| Sri Lanka | 2016 | 89 | 3170 | 51.7 | 47.4 | 15.1 | 69.7 | 37.5 |
| **Sub-Saharan Africa** |  |  |  |  |  |  |  |  |
| Benin | 2016 | 78 | 2477 | 27.0 | 13.3 | 35.1 | 85.9 | 23.2 |
| Liberia | 2017 | 71 | 2164 | 48.6 | 15.9 | 64.2 | 67.4 | 19.7 |
| Mauritania | 2010 | 70 | 1856 | 45.7 | 34.8 | 27.3 | 58.7 | 37.0 |
| Mauritius | 2017 | 84 | 2853 | 53.9 | 42.9 | 24.3 | 82.9 | 40.5 |
| Mozambique | 2015 | 80 | 1724 | 46.1 | 28.5 | 36.4 | 91.0 | 37.4 |
| Namibia | 2013 | 89 | 4267 | 53.6 | 24.7 | 47.5 | 76.5 | 35.8 |
| Seychelles | 2015 | 82 | 2342 | 51.4 | 61.2 | 27.9 | 81.5 | 51.0 |
| Sudan | 2012 | 77 | 1993 | 47.1 | 39.5 | 24.9 | 46.7 | 18.7 |
| Tanzania | 2014 | 87 | 3469 | 50.8 | 60.5 | 15.0 | 64.3 | 19.8 |

**Note.** PE, Physical Education classes. LST, Leisure sitting time. Values expressed in prevalence (95% confidence interval).

**Supplementary table 2.** Prevalence (%) of ≥3h/d of leisure sitting time according the number of weekly PE by country.

| Region/Country | **Number of weekly PE classes** | | | | |
| --- | --- | --- | --- | --- | --- |
|  | 0 day | 1 day | 2 days | 3-4 days | ≥ 5 days |
| **East Asia & Pacific** |  |  |  |  |  |
| Brunei Darussalam | 53.2 (48.7; 57.7) | 56.3 (53.2; 59.4) | 52.9 (47.0; 58.8) | 59.2 (49.3; 68.4) | 60.8 (56.2; 65.3) |
| Cambodia | 12.7 (11.0; 14.7) | 11.7 (9.9; 13.7) | 11.7 (9.0; 15.2) | 10.1 (6.2; 16.0) | 10.9 (7.0; 16.6) |
| Cook Islands | 44.3 (37.4; 51.4) | 48.9 (40.6; 57.2) | 48.5 (38.5; 58.7) | 37.5 (27.7; 48.5) | 41.8 (34.2; 49.9) |
| Fiji | 26.7 (23.4; 30.1) | 26.5 (24.4; 28.7) | 30.0 (25.3; 35.2) | 33.9 (28.6; 39.7) | 39.4 (34.6; 44.5) |
| Indonesia | 20.4 (18.1; 22.9) | 27.6 (26.4; 28.8) | 28.5 (25.8; 31.4) | 29.7 (25.2; 34.6) | 31.5 (28.4; 34.9) |
| Kiribati | 13.0 (9.6; 17.2) | 11.5 (8.8; 14.7) | 16.1 (11.7; 21.7) | 16.1 (11.0; 23.0) | 19.8 (16.0; 24.1) |
| Laos | 24.4 (21.5; 27.6) | 18.8 (17.0; 20.8) | 15.1 (9.2; 23.9) | 28.5 (18.3; 41.4) | 22.5 (17.8; 27.9) |
| Malaysia | 40.6 (38.4; 42.8) | 48.1 (46.9; 49.2) | 45.0 (43.3; 46.7) | 47.3 (44.1; 50.6) | 51.8 (50.2; 53.5) |
| Mongolia | 22.1 (17.2; 27.9) | 47.3 (44.6; 50.1) | 45.6 (43.8; 47.3) | 34.4 (27.4; 42.1) | 39.4 (32.5; 46.6) |
| Myanmar | 16.7 (14.3; 19.4) | 16.4 (14.0; 19.3) | 13.0 (10.3; 16.4) | 17.6 (12.9; 23.5) | 15.4 (11.4; 20.5) |
| Philippines | 45.8 (31.6; 60.7) | 37.9 (17.2; 64.1) | 19.7 (7.6; 42.3) | 32.7 (13.1; 61.0) | 29.5 (15.7; 48.4) |
| Samoa | 21.7 (19.2; 24.5) | 36.2 (34.0; 38.5) | 34.9 (31.3; 38.6) | 36.7 (33.6; 40.0) | 27.7 (25.9; 29.6) |
| Solomon Islands | 22.0 (19.4; 24.9) | 31.6 (24.9; 39.2) | 26.9 (19.0; 36.5) | 42.7 (33.5; 52.3) | 33.2 (27.2; 39.8) |
| Thailand | 21.3 (16.8; 26.6) | 21.5 (16.7; 27.3) | 25.3 (17.2; 35.5) | 24.8 (18.2; 32.8) | 31.8 (26.3; 37.9) |
| Timor-Leste | 44.1 (38.7; 49.5) | 59.4 (56.9; 61.8) | 59.7 (53.5; 65.6) | 63.2 (53.2; 72.2) | 49.6 (41.6; 57.5) |
| Tonga | 10.7 (8.2; 13.8) | 11.7 (10.1; 13.6) | 19.3 (15.5; 23.9) | 29.4 (23.5; 36.0) | 15.0 (12.3; 18.3) |
| Tuvalu | 51.9 (31.1; 72.1) | 27.9 (10.5; 56.0) | 37.6 (20.4; 58.5) | 46.8 (23.8; 71.3) | 60.3 (44.7; 74.1) |
| Vietnam | 19.8 (17.9; 21.9) | 18.8 (15.6; 22.5) | 20.8 (16.5; 25.9) | 28.8 (23.5; 34.7) | 21.9 (18.3; 25.9) |
| Wallis and Futuna | 15.0 (11.7; 19.1) | 13.6 (8.3; 21.3) | 21.1 (12.9; 32.7) | 24.3 (15.1; 36.8) | 19.1 (14.5; 24.8) |
| **Latin America & Caribbean** |  |  |  |  |  |
| Anguilla | 49.3 (40.4; 58.2) | 65.3 (59.7; 70.4) | 44.6 (22.2; 69.5) | 56.9 (40.1; 72.2) | 58.8 (52.8; 64.6) |
| Antigua and Barbuda | 52.9 (46.5; 59.2) | 54.5 (49.5; 59.5) | 55.6 (40.7; 69.6) | 60.8 (45.6; 74.1) | 57.7 (51.0; 64.2) |
| Argentina | 54.6 (52.0; 57.2) | 54.6 (52.6; 56.5) | 57.9 (56.6; 59.3) | 52.1 (48.3; 55.9) | 54.1 (52.5; 55.6) |
| Bahamas | 41.9 (32.1; 52.5) | 53.5 (49.6; 57.3) | 58.2 (49.7; 66.3) | 66.9 (55.0; 77.0) | 58.6 (51.0; 65.7) |
| Barbados | 64.7 (57.8; 70.9) | 64.3 (60.6; 67.8) | 57.7 (43.5; 70.8) | 64.0 (51.8; 74.6) | 69.2 (64.4; 73.5) |
| Belize | 37.8 (34.2; 41.6) | 35.0 (31.7; 38.6) | 37.4 (30.4; 45.0) | 45.6 (36.6; 54.8) | 40.6 (34.5; 46.9) |
| Bolivia | 22.0 (19.2; 25.1) | 30.9 (29.2; 32.6) | 37.2 (32.3; 42.3) | 35.9 (30.9; 41.2) | 32.9 (31.0; 34.8) |
| British Virgin Islands | 66.6 (62.5; 70.5) | 59.2 (54.9; 63.3) | 59.3 (45.2; 72.0) | 60.1 (45.6; 73.1) | 56.8 (51.4; 62.0) |
| Chile | 46.4 (37.9; 55.1) | 56.0 (52.0; 60.0) | 50.7 (44.8; 56.6) | 52.6 (40.7; 64.1) | 55.3 (50.3; 60.3) |
| Costa Rica | 38.6 (34.2; 43.2) | 46.3 (43.4; 49.3) | 41.7 (30.5; 53.9) | 45.6 (35.5; 56.0) | 42.8 (39.3; 46.5) |
| Curaçao | 62.0 (57.6; 66.2) | 61.8 (58.7; 64.7) | 53.4 (43.9; 62.7) | 66.0 (57.7; 73.3) | 63.2 (59.2; 67.0) |
| Dominican Republic | 41.0 (34.4; 47.8) | 46.5 (39.5; 53.6) | 48.9 (41.5; 56.3) | 49.4 (39.2; 59.7) | 51.0 (44.4; 57.5) |
| El Salvador | 33.7 (27.7; 40.3) | 35.6 (31.8; 39.7) | 37.9 (32.1; 44.0) | 38.8 (27.2; 51.8) | 31.8 (28.1; 35.8) |
| Guatemala | 18.0 (12.2; 25.8) | 18.8 (15.7; 22.4) | 27.6 (21.3; 35.0) | 23.6 (14.1; 36.7) | 26.5 (21.5; 32.1) |
| Guyana | 34.7 (31.9; 37.6) | 34.0 (29.8; 38.4) | 36.3 (29.6; 43.5) | 39.6 (31.9; 47.8) | 40.3 (34.1; 46.8) |
| Honduras | 28.4 (21.4; 36.6) | 30.3 (26.6; 34.2) | 30.2 (25.8; 35.0) | 30.2 (20.5; 42.0) | 30.9 (26.5; 35.6) |
| Jamaica | 61.1 (56.5; 65.4) | 53.4 (49.2; 57.5) | 55.1 (43.4; 66.3) | 56.4 (44.9; 67.3) | 53.6 (46.8; 60.3) |
| Panama | 50.7 (47.1; 54.4) | 49.9 (46.6; 53.2) | 43.7 (32.4; 55.6) | 50.6 (40.7; 60.4) | 44.7 (41.3; 48.1) |
| Paraguay | 37.9 (33.4; 42.6) | 35.0 (32.7; 37.4) | 34.6 (27.7; 42.1) | 37.1 (28.7; 46.4) | 28.4 (24.4; 32.8) |
| Peru | 25.9 (20.6; 32.1) | 28.5 (26.7; 30.3) | 36.3 (27.8; 45.7) | 39.8 (20.7; 62.5) | 32.2 (19.8; 47.7) |
| St. Kitts and Nevis | 60.7 (56.3; 64.8) | 56.8 (52.8; 60.7) | 52.5 (39.9; 64.8) | 71.7 (58.8; 81.9) | 60.4 (54.1; 66.2) |
| St. Lucia | 62.5 (58.8; 66.0) | 46.7 (41.9; 51.7) | 56.9 (49.4; 64.1) | 57.8 (49.8; 65.4) | 52.8 (46.7; 58.9) |
| St. Vincent and the Grenadines | 59.9 (56.3; 63.3) | 44.0 (38.2; 49.9) | 48.7 (41.8; 55.6) | 56.2 (49.1; 63.1) | 57.4 (51.1; 63.4) |
| Suriname | 35.5 (30.9; 40.5) | 43.2 (39.8; 46.7) | 39.5 (30.3; 49.5) | 54.4 (44.3; 64.1) | 43.3 (39.1; 47.5) |
| Trinidad and Tobago | 52.8 (49.6; 56.0) | 41.7 (38.0; 45.6) | 46.3 (40.8; 51.8) | 50.9 (44.2; 57.5) | 49.6 (44.5; 54.6) |
| Uruguay | 58.2 (53.2; 63.1) | 52.8 (46.5; 59.0) | 60.6 (58.0; 63.2) | 58.2 (51.8; 64.2) | 57.9 (54.4; 61.3) |
| **Middle East & North Africa** |  |  |  |  |  |
| Algeria | 27.5 (23.7; 31.6) | 25.0 (23.1; 27.1) | 23.1 (17.7; 29.6) | 32.6 (27.0; 38.7) | 32.1 (28.3; 36.2) |
| Bahrain | 53.9 (50.5; 57.3) | 56.7 (53.8; 59.5) | 61.4 (59.0; 63.6) | 63.8 (57.9; 69.2) | 56.9 (54.4; 59.5) |
| Egypt | 30.2 (27.2; 33.3) | 27.8 (24.3; 31.7) | 23.3 (19.6; 27.5) | 38.2 (28.6; 48.9) | 25.2 (20.6; 30.4) |
| Iraq | 23.6 (20.4; 27.1) | 23.6 (19.6; 28.2) | 28.0 (22.8; 33.8) | 39.9 (31.4; 49.0) | 27.9 (23.8; 32.4) |
| Kuwait | 55.2 (51.1; 59.3) | 62.8 (59.6; 65.9) | 69.9 (67.2; 72.6) | 70.4 (64.3; 75.9) | 62.0 (55.2; 68.4) |
| Lebanon | 43.0 (40.5; 45.5) | 45.8 (42.7; 48.9) | 46.4 (41.1; 51.8) | 50.5 (43.4; 57.6) | 45.6 (41.7; 49.5) |
| Morocco | 25.2 (22.5; 28.2) | 32.4 (28.6; 36.4) | 32.3 (29.8; 34.8) | 40.5 (35.2; 46.1) | 34.3 (31.6; 37.1) |
| Oman | 39.5 (36.0; 43.1) | 35.2 (31.9; 38.8) | 41.7 (34.6; 49.2) | 42.7 (38.4; 47.0) | 41.3 (38.4; 44.3) |
| Qatar | 27.1 (23.3; 31.2) | 47.2 (40.4; 54.1) | 58.4 (53.8; 62.7) | 59.8 (52.2; 67.0) | 55.8 (50.3; 61.2) |
| Syrian Arab Republic | 21.7 (18.5; 25.3) | 26.7 (24.4; 29.2) | 20.9 (15.2; 28.2) | 33.2 (24.8; 42.7) | 26.1 (22.5; 30.0) |
| United Arab Emirates | 61.7 (58.9; 64.4) | 57.7 (54.9; 60.5) | 58.9 (55.8; 61.9) | 54.8 (50.3; 59.2) | 60.4 (57.0; 63.6) |
| Yemen. Rep. | 21.5 (19.1; 24.1) | 20.4 (15.9; 25.9) | 31.9 (24.7; 40.1) | 28.4 (21.4; 36.6) | 19.0 (15.6; 23.0) |
| **South Asia** |  |  |  |  |  |
| Afghanistan | 18.0 (15.2; 21.2) | 16.9 (13.4; 21.0) | 31.4 (25.3; 38.3) | 38.9 (33.6; 44.4) | 24.0 (19.6; 28.9) |
| Bangladesh | 15.6 (10.5; 22.8) | 16.6 (11.5; 23.4) | 15.0 (11.9; 18.8) | 20.7 (16.1; 26.2) | 11.1 (8.5; 14.5) |
| Nepal | 9.6 (8.2; 11.3) | 8.1 (6.2; 10.6) | 12.3 (9.3; 16.0) | 13.1 (10.5; 16.3) | 11.3 (9.5; 13.3) |
| Pakistan | 7.0 (6.0; 8.1) | 11.6 (9.2; 14.5) | 8.9 (7.0; 11.2) | 10.7 (7.7; 14.8) | 8.2 (5.8; 11.5) |
| Sri Lanka | 37.4 (34.3; 40.6) | 36.9 (32.3; 41.7) | 37.4 (33.0; 41.9) | 38.0 (33.9; 42.3) | 37.5 (34.0; 41.1) |
| **Sub-Saharan Africa** |  |  |  |  |  |
| Benin | 20.8 (16.2; 26.2) | 21.8 (19.6; 24.2) | 21.2 (15.6; 28.0) | 36.5 (25.2; 49.7) | 33.5 (26.6; 41.3) |
| Liberia | 18.4 (15.6; 21.5) | 16.0 (13.1; 19.4) | 21.9 (16.9; 28.0) | 25.9 (19.9; 33.0) | 22.2 (18.3; 26.8) |
| Mauritania | 36.4 (33.0; 40.0) | 30.9 (26.1; 36.0) | 45.0 (37.1; 53.2) | 42.8 (35.0; 50.9) | 38.1 (33.4; 43.1) |
| Mauritius | 46.9 (42.1; 51.8) | 37.3 (34.6; 40.1) | 41.9 (34.0; 50.3) | 38.7 (31.8; 46.1) | 42.8 (38.6; 47.1) |
| Mozambique | 28.9 (21.9; 37.2) | 35.4 (30.9; 40.1) | 38.5 (33.7; 43.6) | 43.4 (34.9; 52.3) | 40.9 (31.6; 50.8) |
| Namibia | 36.6 (33.5; 39.9) | 29.1 (26.8; 31.5) | 36.8 (31.8; 42.1) | 39.0 (33.2; 45.0) | 44.0 (40.9; 47.1) |
| Seychelles | 50.2 (45.3; 55.1) | 50.2 (47.0; 53.4) | 51.2 (43.8; 58.6) | 57.6 (49.5; 65.3) | 51.3 (47.3; 55.3) |
| Sudan | 16.4 (14.1; 19.0) | 17.8 (14.2; 22.1) | 19.6 (13.0; 28.4) | 31.0 (22.0; 41.8) | 26.2 (18.3; 36.0) |
| Tanzania | 15.3 (13.3; 17.6) | 19.4 (16.1; 23.0) | 23.0 (18.7; 27.9) | 27.5 (23.3; 32.1) | 21.3 (18.5; 24.3) |

**Note.** PE, Physical Education. Values expressed in prevalence (95% confidence interval).

**Supplementary table 3.** Association between the number weekly PE classes and leisure sitting time (≥3 h/d) by country.

| Region/Country | **Number of weekly PE classes** | | | | |
| --- | --- | --- | --- | --- | --- |
|  | 0 day | 1 day | 2 days | 3-4 days | ≥ 5 days |
| **East Asia & Pacific** |  |  |  |  |  |
| Brunei Darussalam | 0.96 (0.87; 1.06) | REF | 0.98 (0.86; 1.11) | 1.06 (0.89; 1.27) | 1.12 (1.02; 1.22) |
| Cambodia | 1.09 (0.88; 1.36) | REF | 1.04 (0.76; 1.41) | 0.88 (0.54; 1.45) | 0.96 (0.61; 1.52) |
| Cook Islands | 0.83 (0.65; 1.05) | REF | 0.98 (0.75; 1.28) | 0.75 (0.54; 1.06) | 0.84 (0.65; 1.08) |
| Fiji | 1.00 (0.86; 1.17) | REF | 1.15 (0.95; 1.38) | 1.28 (1.07; 1.54) | 1.50 (1.29; 1.74) |
| Indonesia | 0.77 (0.68; 1.87) | REF | 1.07 (0.97; 1.19) | 1.05 (0.89; 1.24) | 1.15 (1.03; 1.29) |
| Kiribati | 1.12 (0.76; 1.65) | REF | 1.40 (0.94; 2.10) | 1.38 (0.88; 2.17) | 1.70 (1.22; 2.36) |
| Laos | 1.28 (1.09; 1.50) | REF | 0.81 (0.50; 1.33) | 1.51 (0.99; 2.28) | 1.20 (0.94; 1.53) |
| Malaysia | 0.84 (0.79; 1.89) | REF | 0.93 (0.89; 0.98) | 1.00 (0.93; 1.07) | 1.09 (1.05; 1.14) |
| Mongolia | 0.49 (0.38; 1.63) | REF | 0.99 (0.93; 1.06) | 0.78 (0.62; 0.97) | 0.89 (0.73; 1.07) |
| Myanmar | 0.99 (0.79; 1.24) | REF | 0.81 (0.60; 1.08) | 1.05 (0.75; 1.48) | 0.93 (0.67; 1.30) |
| Philippines | 0.86 (0.47; 1.56) | REF | 0.76 (0.26; 2.21) | 0.90 (0.33; 2.46) | 0.77 (0.37; 1.61) |
| Samoa | 0.60 (0.53; 1.69) | REF | 0.97 (0.86; 1.10) | 1.01 (0.91; 1.13) | 0.77 (0.70; 0.84) |
| Solomon Islands | 0.66 (0.51; 1.85) | REF | 0.84 (0.56; 1.25) | 1.28 (0.93; 1.76) | 1.01 (0.75; 1.35) |
| Thailand | 0.99 (0.71; 1.39) | REF | 1.16 (0.75; 1.79) | 1.18 (0.80; 1.73) | 1.51 (1.11; 2.04) |
| Timor-Leste | 0.76 (0.67; 1.86) | REF | 1.06 (0.95; 1.19) | 1.08 (0.92; 1.26) | 0.89 (0.75; 1.05) |
| Tonga | 0.90 (0.67; 1.21) | REF | 1.63 (1.25; 2.11) | 2.48 (1.91; 3.22) | 1.28 (0.99; 1.64) |
| Tuvalu | 1.68 (0.68; 1.13) | REF | 1.50 (0.56; 4.00) | 1.59 (0.58; 4.34) | 2.27 (0.95; 5.45) |
| Vietnam | 1.04 (0.84; 1.28) | REF | 1.09 (0.82; 1.46) | 1.50 (1.15; 1.96) | 1.15 (0.89; 1.47) |
| Wallis and Futuna | 1.06 (0.63; 1.79) | REF | 1.46 (0.76; 2.79) | 1.90 (0.98; 3.68) | 1.34 (0.79; 2.29) |
| **Latin America & Caribbean** |  |  |  |  |  |
| Anguilla | 0.74 (0.61; 3.90) | REF | 0.69 (0.39; 1.23) | 0.90 (0.66; 1.22) | 0.89 (0.79; 1.02) |
| Antigua and Barbuda | 0.94 (0.81; 3.10) | REF | 1.04 (0.79; 1.37) | 1.12 (0.87; 1.44) | 1.06 (0.92; 1.23) |
| Argentina | 1.00 (0.94; 3.06) | REF | 1.06 (1.02; 1.11) | 0.96 (0.88; 1.04) | 1.00 (0.95; 1.04) |
| Bahamas | 0.82 (0.64; 3.05) | REF | 1.12 (0.95; 1.31) | 1.26 (1.05; 1.51) | 1.13 (0.97; 1.30) |
| Barbados | 0.95 (0.85; 3.07) | REF | 0.86 (0.67; 1.10) | 0.98 (0.81; 1.17) | 1.08 (0.99; 1.18) |
| Belize | 1.08 (0.94; 3.24) | REF | 1.05 (0.85; 1.31) | 1.30 (1.05; 1.63) | 1.17 (0.97; 1.40) |
| Bolivia | 0.71 (0.62; 3.82) | REF | 1.21 (1.05; 1.41) | 1.15 (0.99; 1.35) | 1.07 (0.99; 1.16) |
| British Virgin Islands | 0.99 (0.89; 3.09) | REF | 1.04 (0.81; 1.32) | 0.95 (0.74; 1.22) | 0.97 (0.87; 1.09) |
| Chile | 0.81 (0.66; 3.99) | REF | 0.91 (0.80; 1.05) | 0.92 (0.72; 1.17) | 0.99 (0.88; 1.11) |
| Costa Rica | 0.82 (0.72; 3.94) | REF | 0.91 (0.68; 1.22) | 0.98 (0.78; 1.24) | 0.93 (0.84; 1.03) |
| Curaçao | 0.97 (0.89; 3.06) | REF | 0.87 (0.72; 1.05) | 1.06 (0.93; 1.20) | 1.01 (0.94; 1.09) |
| Dominican Republic | 0.88 (0.70; 3.10) | REF | 1.05 (0.85; 1.31) | 1.09 (0.84; 1.40) | 1.11 (0.91; 1.35) |
| El Salvador | 0.95 (0.77; 3.18) | REF | 1.07 (0.88; 1.30) | 1.10 (0.78; 1.55) | 0.90 (0.76; 1.06) |
| Guatemala | 0.96 (0.63; 3.45) | REF | 1.45 (1.07; 1.97) | 1.28 (0.77; 2.14) | 1.41 (1.08; 1.84) |
| Guyana | 0.99 (0.85; 3.16) | REF | 1.07 (0.85; 1.35) | 1.15 (0.90; 1.46) | 1.19 (0.97; 1.46) |
| Honduras | 0.95 (0.70; 3.27) | REF | 1.00 (0.82; 1.22) | 1.00 (0.68; 1.47) | 1.02 (0.84; 1.24) |
| Jamaica | 1.03 (0.92; 3.15) | REF | 0.98 (0.77; 1.23) | 1.04 (0.83; 1.29) | 1.01 (0.87; 1.17) |
| Panama | 0.99 (0.90; 3.09) | REF | 0.90 (0.68; 1.20) | 1.05 (0.85; 1.29) | 0.91 (0.82; 1.00) |
| Paraguay | 1.09 (0.95; 3.26) | REF | 1.01 (0.81; 1.26) | 1.09 (0.85; 1.40) | 0.85 (0.72; 1.00) |
| Peru | 0.91 (0.72; 3.15) | REF | 1.29 (0.99; 1.67) | 1.37 (0.79; 2.40) | 1.13 (0.72; 1.77) |
| St. Kitts and Nevis | 1.05 (0.95; 3.16) | REF | 0.97 (0.76; 1.25) | 1.31 (1.09; 1.57) | 1.08 (0.96; 1.22) |
| St. Lucia | 1.20 (1.06; 3.36) | REF | 1.18 (1.00; 1.40) | 1.20 (1.01; 1.43) | 1.11 (0.95; 1.29) |
| St. Vincent and the Grenadines | 1.30 (1.12; 3.51) | REF | 1.09 (0.90; 1.33) | 1.26 (1.05; 1.52) | 1.30 (1.09; 1.54) |
| Suriname | 0.83 (0.71; 3.97) | REF | 0.92 (0.71; 1.19) | 1.27 (1.04; 1.55) | 1.01 (0.89; 1.14) |
| Trinidad and Tobago | 1.14 (1.02; 3.28) | REF | 1.12 (0.97; 1.31) | 1.19 (1.02; 1.40) | 1.19 (1.04; 1.37) |
| Uruguay | 1.10 (0.95; 3.27) | REF | 1.16 (1.02; 1.31) | 1.13 (0.96; 1.33) | 1.11 (0.98; 1.27) |
| **Middle East & North Africa** |  |  |  |  |  |
| Algeria | 1.15 (0.98; 4.35) | REF | 0.97 (0.74; 1.26) | 1.34 (1.10; 1.62) | 1.31 (1.13; 1.51) |
| Bahrain | 0.93 (0.86; 4.01) | REF | 1.05 (0.98; 1.11) | 1.09 (0.99; 1.21) | 1.01 (0.95; 1.08) |
| Egypt | 1.20 (1.02; 4.42) | REF | 0.74 (0.59; 0.92) | 1.31 (0.97; 1.77) | 0.87 (0.69; 1.09) |
| Iraq | 1.05 (0.83; 4.32) | REF | 1.19 (0.91; 1.55) | 1.70 (1.27; 2.27) | 1.18 (0.93; 1.49) |
| Kuwait | 0.88 (0.80; 4.96) | REF | 1.14 (1.07; 1.22) | 1.14 (1.04; 1.26) | 1.02 (0.90; 1.15) |
| Lebanon | 0.93 (0.85; 4.01) | REF | 1.04 (0.91; 1.19) | 1.12 (0.95; 1.31) | 1.04 (0.94; 1.16) |
| Morocco | 0.81 (0.69; 4.95) | REF | 0.98 (0.85; 1.13) | 1.21 (1.01; 1.44) | 1.04 (0.90; 1.19) |
| Oman | 1.09 (0.95; 4.25) | REF | 1.15 (0.94; 1.41) | 1.18 (1.02; 1.36) | 1.16 (1.03; 1.31) |
| Qatar | 0.57 (0.46; 4.69) | REF | 1.19 (1.01; 1.40) | 1.23 (1.02; 1.49) | 1.17 (0.98; 1.39) |
| Syrian Arab Republic | 0.84 (0.70; 4.00) | REF | 0.80 (0.58; 1.11) | 1.27 (0.95; 1.70) | 0.97 (0.82; 1.15) |
| United Arab Emirates | 1.04 (0.98; 4.11) | REF | 1.06 (0.99; 1.13) | 0.99 (0.90; 1.09) | 1.07 (1.00; 1.15) |
| Yemen. Rep. | 0.92 (0.70; 4.21) | REF | 1.56 (1.11; 2.20) | 1.38 (0.95; 1.98) | 0.95 (0.69; 1.29) |
| **South Asia** |  |  |  |  |  |
| Afghanistan | 1.04 (0.79; 6.38) | REF | 1.87 (1.38; 2.55) | 2.32 (1.77; 3.02) | 1.39 (1.04; 1.88) |
| Bangladesh | 0.89 (0.52; 6.54) | REF | 0.93 (0.61; 1.43) | 1.24 (0.80; 1.91) | 0.65 (0.41; 1.02) |
| Nepal | 1.18 (0.86; 6.61) | REF | 1.49 (1.02; 2.18) | 1.56 (1.10; 2.20) | 1.35 (0.98; 1.85) |
| Pakistan | 0.62 (0.47; 6.81) | REF | 0.78 (0.56; 1.08) | 0.92 (0.62; 1.38) | 0.70 (0.46; 1.06) |
| Sri Lanka | 1.00 (0.86; 6.17) | REF | 1.04 (0.87; 1.24) | 1.05 (0.89; 1.24) | 1.02 (0.87; 1.19) |
| **Sub-Saharan Africa** |  |  |  |  |  |
| Benin | 0.95 (0.73; 7.24) | REF | 0.95 (0.70; 1.30) | 1.67 (1.18; 2.37) | 1.55 (1.21; 1.97) |
| Liberia | 1.16 (0.90; 7.49) | REF | 1.40 (1.01; 1.93) | 1.63 (1.18; 2.24) | 1.40 (1.07; 1.85) |
| Mauritania | 1.17 (0.97; 7.41) | REF | 1.44 (1.14; 1.84) | 1.36 (1.07; 1.75) | 1.23 (1.00; 1.51) |
| Mauritius | 1.22 (1.07; 7.38) | REF | 1.12 (0.91; 1.38) | 1.02 (0.83; 1.24) | 1.14 (1.01; 1.29) |
| Mozambique | 0.81 (0.60; 7.10) | REF | 1.09 (0.91; 1.31) | 1.23 (0.97; 1.56) | 1.17 (0.90; 1.54) |
| Namibia | 1.27 (1.13; 7.43) | REF | 1.27 (1.08; 1.49) | 1.35 (1.14; 1.60) | 1.52 (1.36; 1.69) |
| Seychelles | 0.99 (0.88; 7.11) | REF | 1.07 (0.92; 1.25) | 1.19 (1.02; 1.38) | 1.04 (0.94; 1.15) |
| Sudan | 0.97 (0.73; 7.28) | REF | 1.12 (0.72; 1.76) | 1.80 (1.22; 2.67) | 1.49 (0.99; 2.23) |
| Tanzania | 0.79 (0.63; 7.99) | REF | 1.18 (0.91; 1.54) | 1.42 (1.12; 1.80) | 1.11 (0.89; 1.39) |

**Note.** REF, Reference group. PE, Physical Education. Values expressed in prevalence ratio and 95% confidence interval. Adjusted for age, sex and food insecurity.

**Supplementary table 4.** Association between the number of PE classes and leisure sitting time (≥5 h/d) according by region and income.

|  | **Number of weekly PE classes** | | | | |
| --- | --- | --- | --- | --- | --- |
|  | 0 day | 1 day | 2 days | 3-4 days | ≥ 5 days |
| **Total** | 1.06 (1.02; 1.12) | REF | 1.06 (1.02; 1.11) | 1.32 (1.23; 1.41) | 1.29 (1.22; 1.36) |
| **Region** |  |  |  |  |  |
| East Asia & Pacific | 0.96 (0.87; 1.07) | REF | 1.04 (0.94; 1.14) | 1.26 (1.07; 1.51) | 1.37 (1.20; 1.57) |
| Latin America &  Caribbean | 1.09 (1.04; 1.16) | REF | 1.06 (1.01; 1.12) | 1.20 (1.10; 1.30) | 1.18 (1.12; 1.25) |
| Middle East & North Africa | 1.10 (0.96; 1.27) | REF | 1.09 (1.00; 1.37) | 1.44 (1.23; 1.70) | 1.23 (1.12; 1.37) |
| South Asia | 1.14 (0.93; 1.40) | REF | 1.08 (0.84; 1.38) | 1.80 (1.15; 2.83) | 1.39 (0.95; 2.05) |
| Sub-Saharan Africa | 1.15 (0.99; 1.33) | REF | 1.15 (1.03; 1.11) | 1.49 (1.24; 1.79) | 1.61 (1.34. 1.94) |
| **Income** |  |  |  |  |  |
| Low | 1.08 (0.86; 1.35) | REF | 1.24 (0.90; 1.71) | 2.01 (1.62; 2.51) | 1.60 (1.20; 2.14) |
| Lower-middle | 1.05 (0.94; 1.17) | REF | 1.11 (1.01; 1.22) | 1.44 (1.25; 1.67) | 1.35 (1.22; 1.49) |
| Upper-middle | 1.13 (1.05; 1.22) | REF | 1.03 (0.98; 1.08) | 1.25 (1.11; 1.41) | 1.33 (1.20; 1.48) |
| High | 1.03 (0.96; 1.12) | REF | 1.04 (0.99; 1.11) | 1.16 (1.07; 1.26) | 1.16 (1.09; 1.23) |

**Note.** REF, Reference group. Values expressed in prevalence ratio and 95% confidence interval. Adjusted for age, sex and food insecurity.
